# Exploring the association between microbiota and behaviour in suckling piglets

**DOI:** 10.1038/s41598-022-16259-3

**Published:** 2022-07-19

**Authors:** R. Choudhury, A. Middelkoop, J. E. Bolhuis, M. Kleerebezem

**Affiliations:** 1 Host-Microbe Interactomics Group, Department of Animal Sciences, Wageningen University & Research, P.O. Box 338, 6700 AH Wageningen, The Netherlands; 2 Adaptation Physiology Group, Department of Animal Sciences, Wageningen University & Research, P.O. Box 338, 6700 AH Wageningen, The Netherlands

**Keywords:** Applied microbiology, Microbiome

## Abstract

It is increasingly recognised that the microbes residing in the gastrointestinal tract can influence brain physiology and behaviour, via the microbiota–gut–brain axis. Here, we made a first explorative evaluation at the association between the gut microbiota and behaviour in suckling piglets. 16S microbiota profiling information was obtained from two independent replicate experiments at 2 and 4 weeks of age. Piglets underwent a backtest to assess their personality or coping style at 2 weeks of age, and were subjected to a combined open field and novel object test at 3.5 weeks of age, recording anxiety-related and exploratory behaviour. The number of squeals vocalised during the open field test was associated with microbial groups such as *Coprococcus 3* and *CAG-873*, whereas in the novel object test, explorative behaviour was significantly associated with microbial genera like *Atopobium* and *Prevotella.* Overall, this study explores the microbiota-behavioural relation by employing multivariate analysis and exemplifies the importance of individualised analyses when evaluating such relationships.

## Introduction

Increasing evidence indicates that the impact of gut microbiota extends beyond the gut, as it plays a crucial role in the communication between the intestine and central nervous system (CNS), also known as the microbiota–gut–brain axis^1–3^. The mediators of this communication include short-chain fatty acids, neurotransmitters, immune system modulators, hormones, as well as the vagus nerve, which are all known to be affected by microbial metabolism^4–6^. During early life the intestinal microbiota is dynamic and rapidly evolving, which coincides with important developmental processes in the brain. This overlapping timeline of early life microbiota and brain development could provide a “window of opportunity” for influencing CNS development and function, via microbial modulation^7^. The microbiota–gut–brain axis consists of bidirectional communication, with the microbiota shown to play a role in neurodevelopment (from early life to adulthood) and behaviour by influencing neural processes such as myelination, neurogenesis, neurotransmission and development of the hypothalamic–pituitary–adrenal axis (HPA)^3,7^. On the other hand, the brain regulates intestinal functions (e.g. motility, secretion and mucin production) as well as immune functions (e.g. modulation of cytokine production)^1^ in the gastro-intestinal tract. However, the underlying mechanisms of the bidirectional communication between the gut and the brain are not fully understood.

Recent advancement of sequencing technology has allowed exploration of the microbiome and its neuroactive potential in the context of stress, anxiety and depression-related behaviour^4^. Animal models can play an important role in understanding the underlying mechanisms via which early life experiences affect later life health. Mostly, gnotobiotic or germ free rodent models have been employed to get insight into the mechanism of the microbiota–gut–brain communication, using probiotics, antibiotics, drugs or faecal transplantation^8,9^. However, compared to humans, the CNS development is substantially different in rodent species as they have a less developed brain at birth^10^, with maximum development occurring postnatally. On the other hand, pigs and humans exhibit striking similarity with respect to their physiology, brain development and gastrointestinal function^11,12^, including an analogous “window of brain development” at birth as well as having a gyrencephalic brain^10^. Apart from its value as a translational model for human development, disease and the underlying processes, the understanding of early-life development in pigs is also valuable in the context of veterinary and animal sciences. However, there are only a handful of studies^13–15^ evaluating the relationship between microbial communities and behavioural characteristics in pigs, reflecting the putative communication with the gut and the brain.

It is well known that microbiota differs among individuals, and therefore needs to be explored as an individualised mechanism to explain differences in behaviour, which remains virtually unexplored^7^. The present study explores the association between the microbiota composition and behaviour of individual suckling piglets in challenging situations (which could reflect their personality) using multivariate approaches, aiming to take a step towards better understanding of the biological relevance of microbiome variation in behaviour.

## Results

**Figure 1:**
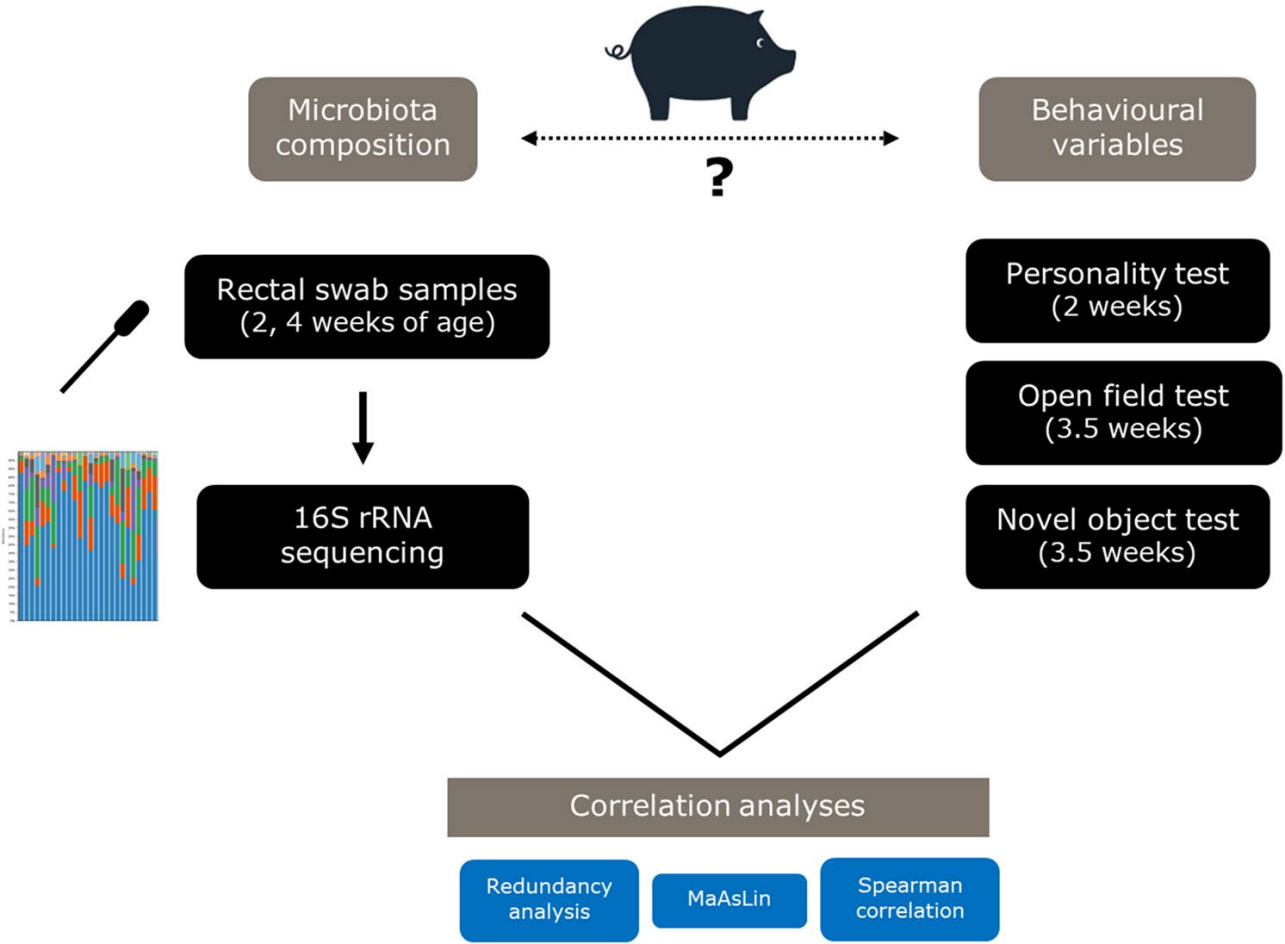
Schematic study design to assess association between the microbiota and behaviour. Microbiota and behavioural data were obtained from two independent experiments. Rectal swabs were collected at day 15 (2 weeks of age) and day 28 (4 weeks of age) pre-weaning for microbiota analysis. Personality (or back) test and Anxiety test (open field test, novel object test) were performed at day 15 (2 weeks of age) and day 25 (3.5 weeks of age) respectively, in suckling piglets.

Group based analyses employing categorical variables such as treatment, gender and pen did not reveal any associations with the behavioural parameters. To understand individual differences in behavioural traits, we explored associations between microbiota composition and behavioural observations at an individual level (Fig. 1). Correlation analyses were performed by combining data from two replicate experiments. The microbiota composition data generated 1,941,012 number of reads after quality filtering, with a mean sequencing depth of 20,128 ± 5905 reads per sample.

**Figure 2:**
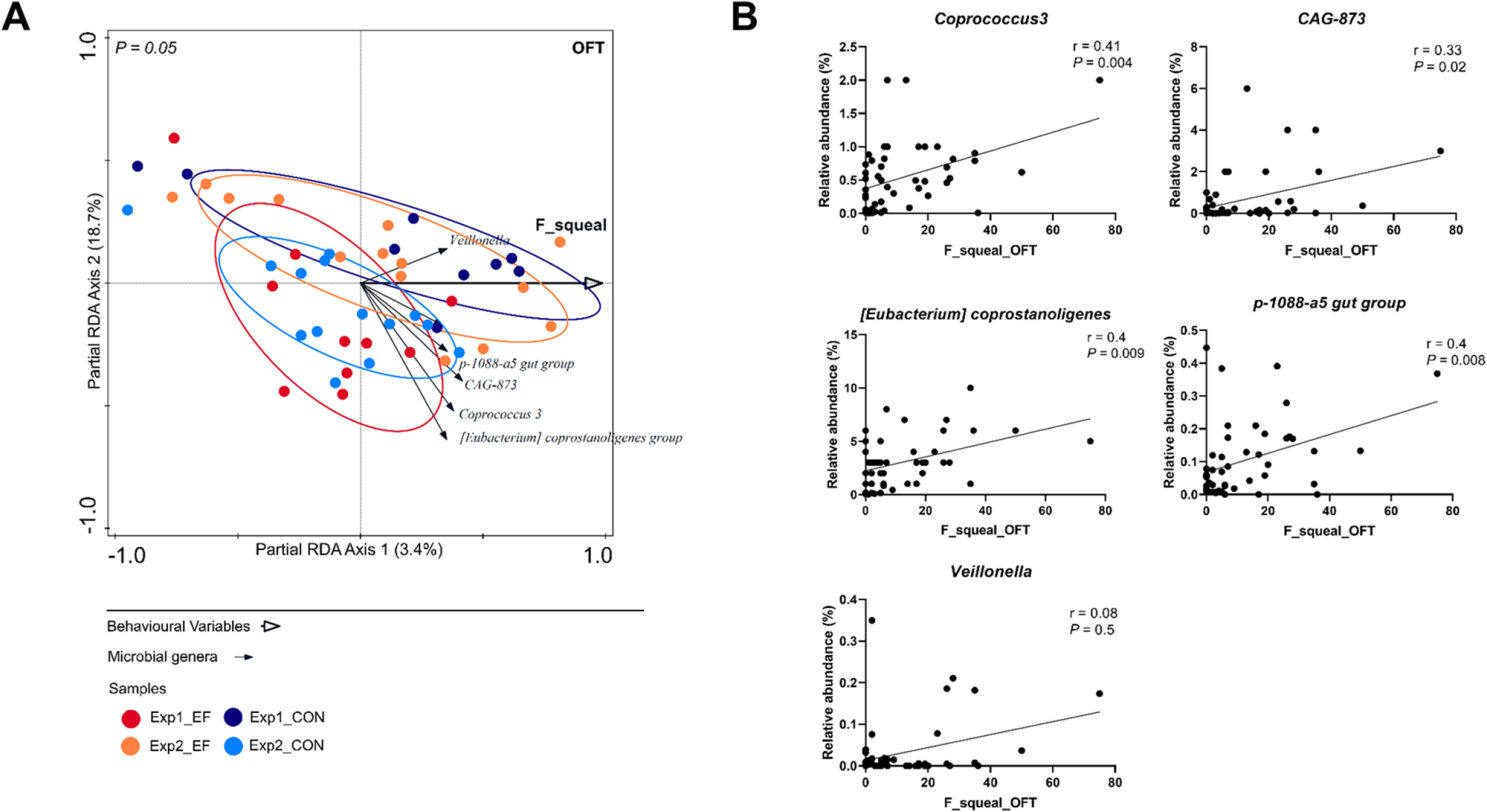
Assessing microbial associations of individual behavioural variables in open field test (OFT). (**A**) Partial redundancy analysis (pRDA) of the frequency of squeals (F_squeal_OFT), corrected for experiment and treatment. In the pRDA triplot, individual animals are indicated in coloured balls belonging to either one of the experiments and treatment group. Microbial groups having (1) RDA response score ≥ 0.35 as well as (2) minimum 0.1% relative abundance in at least 10% of the samples, are visualised in the triplot. The perpendicular distance between microbes and environmental variable axes in the plot reflects their correlations. The smaller the distance, the stronger the correlation. (**B**) Spearman correlation of individual pRDA identified microbes with the frequency of squeals.

We analysed four backtest variables with the microbiota composition at 2 weeks of age by redundancy analysis (RDA), but did not find a significant correlation (*P* = 0.66; Supplementary Fig. 1B). To investigate fear-related as well as explorative behaviour in suckling piglets, a combined OFT and NOT was performed at 3.5 weeks of age. In the OFT, the frequency of squeals was significantly associated with the microbiota composition (Fig. 2A), displaying the strongest correlation in the RDA plot (longest arrow; Supplementary Fig. 1C). The frequency of squeals during the OFT was positively associated with the abundance of microbial genera such as *Coprococcus 3, CAG-873, Eubacterium coprostanoligenes, p-1088-a5 gut group* and *Veillonella.* Individual Spearman correlation analysis re-affirmed the RDA correlation observations, except for *Veillonella* whose association appeared to be insignificant (Fig. 2B). Other behavioural variables in the OFT did not relate to the microbiota composition.

**Figure 3:**
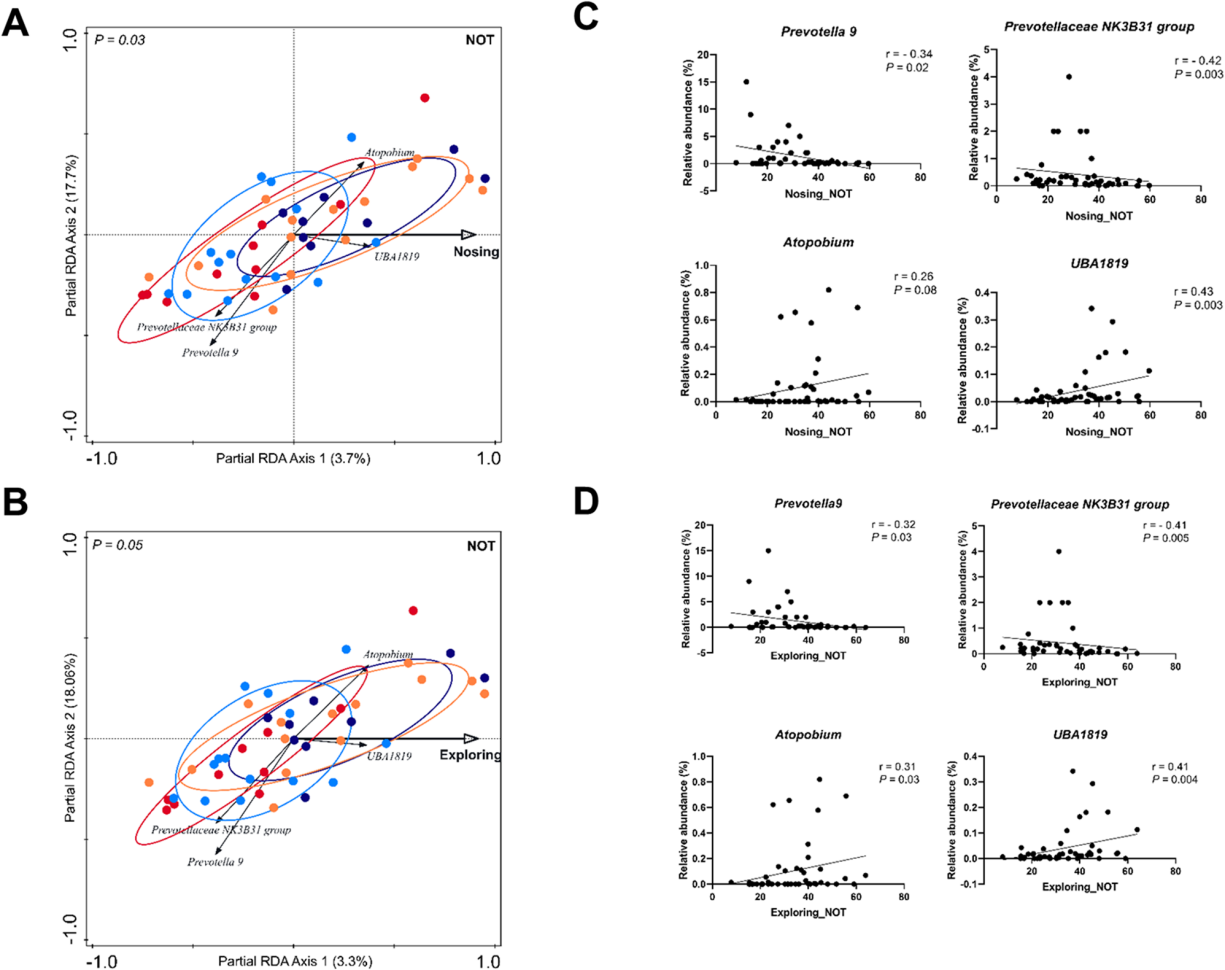
Assessing microbial associations of individual behavioural variables in novel object test (NOT). (**A**) Partial redundancy analysis (pRDA) of the nosing behaviour (% of time spent nosing) during the novel object test, corrected for experiment and treatment. (**B**) pRDA of the exploring behaviour (nosing + rooting: both walls and floor; % of time spent exploring), corrected for experiment and treatment. In the pRDA triplot, individual animals are indicated in coloured balls belonging to either one of the experiments and treatment group. Microbial groups having (1) RDA response score ≥ 0.35 as well as (2) minimum 0.1% relative abundance in at least 10% of the samples, are visualised in the triplot. The perpendicular distance between microbes and environmental variable axes in the plot reflects their correlations. The smaller the distance, the stronger the correlation. (**C**) Spearman correlation of pRDA identified microbes with nosing behaviour. (**D**) Spearman correlation of pRDA identified microbes with exploring behaviour.

Similarly, NOT variables were assessed to evaluate whether an animal’s reaction to a novel object was associated with its microbiota composition (Supplementary Fig. 1D). We found six observations linked to exploration behaviour, having significant associations with microbiota. Behavioural parameters such as nosing (walls + floor), exploring (nosing + rooting of walls and floor), walls (nosing + rooting walls), nosing floor, nosing walls as well as exploring the novel object were found significant (*P* < 0.1; Fig. 3A,B; Supplementary Fig. 2A–E). However, no significant correlation was found between ‘latency to touch the novel object’ and microbiota, that is related to the fear response of the pig (Supplementary Fig. 2F). In most of the exploration-related variables, similar microbes were found associated at both genus and OTU level, supporting the inter-relatedness of the behavioural parameters used (data not shown). Notably, piglets spent much more time displaying nosing behaviour relative to rooting behaviour, indicating that the overall exploration behaviour is predominantly directed by the nosing behaviour (Supplementary Table 2). Both nosing and total exploration behaviour negatively correlated with the abundance of *Prevotella 9* and *Prevotellaceae NK3B31 group* and positively with microbial groups like *Atopobium* and *UBA1819* (Fig. 3A,B). Individual Spearman correlation analysis found moderate, yet significant correlations between the identified microbial groups and nosing/exploring behaviour (Fig. 3C,D). Notably, *Eubacterium coprostanoligenes* (5 OTUs), *UBA1819* (1 OTU) and *Atopobium* (1 OTU) were associated with respective behavioural observations at the OTU level. Further, most of these identified behavioural parameters were interrelated, indicated by similar (quartile) distribution of individual piglets (based on nosing behaviour) with other behavioural parameters (Supplementary Fig. 1E). Intriguingly, ‘explore novel object’ showed an opposite association with *Prevotellaceae NK3B31 group* compared to other exploration-related behaviour like ‘nosing’ and ‘exploring’ (Supplementary Fig. 2), which is also evident from their negative correlation (Supplementary Fig. 3) as well as the inverse distribution of individual piglets in ‘explore novel object’ behaviour when grouped by nosing behaviour (Supplementary Fig. 1E).

To compare results obtained by redundancy analyses, MaAsLin analysis was performed using both default as well as congruent settings. Using the MaAsLin default settings that are more stringent than those we employed in RDA analyses, we did not identify any significant microbial associations (Benjamini–Hochberg FDR corrected *P* value < 0.05). However, upon relaxing the correction method and employing microbiota cut-off settings analogous to the RDA analyses, the MaAsLin analyses revealed various microbial groups including several found by RDA, to be associated with behavioural parameters albeit with varying degree of strength (Supplementary Table 3).

## Discussion

The association between microbiota and behaviour is increasingly studied and accumulating evidence indicates that the gut microbiota can influence animal behaviour^3,5,8,13,15^, although the underlying mechanisms largely remain to be deciphered. We previously investigated the influence of diet on gut microbiota and host mucosa development during early life in pigs^16,17^. In the present study we explored whether the intestinal microbiota composition in pigs is associated with host behavioural traits at an individual animal level (independent of dietary treatment), by employing multivariate correlation analyses. This study aimed to tentatively reveal associations between the intestinal microbiota composition and piglet behaviour in a test for coping style, (i.e. a personality trait), as well as anxiety- and exploration-related behavioural parameters in an open field-novel environment test. These associations between microbiota composition and behaviour of suckling piglets in challenging situations have, to the best of our knowledge, not been investigated before.

Previous studies stated that behavioural responses during the ‘backtest’ are indicative of individual coping responses to environmental stressors^18,19^, often classifying them into high and low-resisting animals based on the latencies and frequencies of struggling and vocalising^20^. High-resisting animals seem more likely to adopt an active coping style, whereas low-resisting animals that remain immobile and silent during the backtest adopt a passive, or reactive coping style^18^. In this study, the coping style of suckling piglets did not have any association with the corresponding microbiota composition at 2 weeks of age. A possible explanation for this might be that the microbiota colonisation in the first few weeks of life, is usually dynamic and apparently chaotic leading to relatively high individual variation, suggesting that an extended sample size would probably be required to assess microbial association with parameters obtained during the backtest. An alternative could be to perform the backtest at a later age, for instance just before weaning, when the microbiota has relatively stabilised, and investigate whether there is a relation between coping style and microbiota.

Prior studies indicate that vocalisations can reflect an animal’s (pig) emotional and/or physiological state^21^. Low-pitched vocalisations (grunts) might be used to maintain social contact, while high-pitched vocalisations, particularly squeals and screams, could relate to distress or anxiety, especially when the animal is in isolation^21–23^. Suckling piglets were subjected to a combined OFT and NOT to evaluate their anxiety- and exploration-related responses in a novel environment. In the present study, we observed that the number of squeals uttered by piglets had a significant association with their microbiota. However, we found no significant microbiota correlation with the frequency of long grunts, which has been previously reported to be strongly associated (r = 0.75, *P* < 0.05) with the frequency of squeals in a similar test in adult pigs^24^. In the present study we only detect a minimal correlation between these two behaviour (r = 0.3, *P* = 0.07; Supplementary Fig. 3).

Exploratory behaviour in a challenging situation can reflect anxiety, with lower levels of exploration indicative of more fearfulness^25,26^. During the NOT in our study, the piglets displayed higher frequency of squeals, spent more time on standing alert and less time exploring the test arena (especially nosing floor), compared to the OFT (Supplementary Table 2), that was not fully compensated by the time spent on ‘exploration of novel object’ during the NOT. This suggests that the piglets were more alert/fearful in the NOT, possibly indicating ‘exploration during the NOT’ as a better reflection of (lack of) fear/anxiety compared to OFT. This is in line with a previous study^27^ that found associations among platelet serotonin, brain serotonin and behaviour during the NOT phase, but not during the novel environment (or OFT) phase. Another study^28^ demonstrated the effect of an anxiolytic drug on pig behaviour, only in an elevated plus maze but not in a novel environment test. This potentially suggests that exposure to a novel environment alone (OFT) was not particularly fear-provoking for pigs, as their level of fearfulness is more evident in a relatively challenging situation (e.g., NOT), which is also coherent with our findings with more associations observed when the novel object was added to the novel environment.

In our study, *Coprococcus* showed a moderate positive correlation with the frequency of squeals in the OFT, however, this is contrary to previous mice/human studies that showed an inverse correlation with stress^29^ or depression^4^. In addition, we found microbial groups like *Atopobium* and *Prevotella/*Prevotellaceae to be positively and negatively associated with exploration behaviour, respectively. This is consistent with a previous mice study on maternally separation which reported that ‘anxious’ mice have a reduced *Atopobium* abundance^30^. Moreover, our results are also in agreement with existing literature reporting positive associations between *Prevotella* and depression/anxiety in rodents or humans^31,32^, which would be in line with ‘less exploring’ behaviour in the present study. Although these parallel findings are intriguing, it is important to note that comparisons of microbiota-behavioural associations across different animal species are challenging, as we might be potentially compare apples and oranges. This is because both behavioural observational scores as well as the coinciding development of the central nervous systems and intestinal microbiota during early life can be potentially very different across species. In a previous study^4^, apart from an inverse relation with depression, the *Coprococcus* relative abundance was proposed to coincide with abundance of genes associated with dopamine biosynthesis in the microbiota. The paper further assembled a catalogue or framework called gut brain modules (GBMs), that represent ‘neuroactive potential’ of microbes annotated for function, pathway, structure and potential to cross the intestinal epithelium and the blood–brain barrier. The microbial groups identified in the present study, such as *Coprococcus, Eubacterium* and *Prevotella* are described in the GBMs having microbial neuroactive potential, which is noteworthy.

Interestingly, ‘exploring novel object’ revealed an opposite trend of association with the intestinal microbiota, possibly reflecting the ambiguity of ‘general exploration’ behaviour and ‘novel-object-oriented’ behaviour and their connection to an animal’s level of fear. As mentioned above, squeal (‘high-pitch’ vocalisation) and exploratory behaviour directed at the environment have previously been positively and negatively linked with anxiety in piglets respectively. However in the present study, we did not observe a significant negative correlation between the frequency of squeals/latency to explore NO (fearfulness) and nosing (exploratory) behaviour (Supplementary Fig. 3). In other words, we did not identify an ‘anxious’ group of piglets displaying both high squeal behaviour (OFT) and low exploration behaviour (NOT). A possible explanation for this apparent discrepancy can be because pigs are opportunistic omnivores and relatively neophilic (unlike rodents), with ‘novelty’ not only perceived as a potential threat but also as an opportunity. Besides, pigs have a natural instinct or ‘behavioural need’ to explore, which is extremely limited in their barren, stimulus-poor housing (not enriched). Thus, during their exposure to novel environment (e.g., OFT), social isolation might be the most stressful part and the test could well be experienced as an outlet for exploring the environment. Hence, their behaviour can be reflecting a mixture of fear (fulness), motivation to explore (“curiosity”), and motivation to join their pen mates and mom (for warmth and food). Another point to recognise is our lack of understanding of the complexity of the (animal) behavioural phenotype. For instance, although the open-field test has been widely used to assess pig emotion, there are insufficient evidence to justify its validity as a test for fearful or anxious behaviour in pigs^33^. Apart from the lack of consensus in assessing and interpreting individual behavioural parameters, it is even more complex to interpret the overall open field behaviour in pigs^26^. For example, ‘freeze’ behaviour (or standing alert) in a novel environment, has been differently interpreted by previous studies, where it has been considered to reflect either a ‘fear response’^34^ or as a ‘state of arousal’^35^ in which the animal orients itself to toward the stimulus to investigate, and likely reflects both. Furthermore, suckling piglets are hardly tested, so we do not know whether their open field behaviour reflects the same as weaned/adult pigs as reported in other studies. Therefore, the ambiguities in behavioural interpretations has to be taken into account when inferring the overall biological consequence of correlations between microbiota and a single observed behavioural parameter. Nevertheless, the porcine microbiota-behavioural associations observed in this study deserve further evaluations, to establish their biological relevance and underlying mechanism(s).

Taken together, this study provides a first explorative evaluation into the association of early-life behaviour and microbiota in suckling piglets. It also demonstrates the importance of individual analysis when evaluating behavioural and microbiota associations, both of which are individual-specific traits. Notably, most behavioural tests have been developed and standardised for post-weaning or growing pigs, which are more mature as compared to the suckling (still developing) piglets employed in our study. For future studies in young piglets, the behavioural tests might need to be adjusted and verified accordingly to ensure that they adequately reflect anxiety and/or explorative behaviour. Although there is a lack of similar studies reported in the literature which disables the comparative analysis of the results obtained in this study, the approaches used in this study may inspire the design of new experiments and strategies to evaluate the role of the microbiota–gut–brain axis in behavioural development of young animals.

## Methods

### Study design

The Animal Care and Use committee of Wageningen University & Research (Wageningen, The Netherlands) approved the protocol of the experiment (AVD104002016515). The protocol is in accordance with the Dutch law on animal experimentation, which complies with the European Directive 2010/63/EU on the protection of animals used for scientific purposes. The experiments were carried out in compliance with the ARRIVE guidelines (https://arriveguidelines.org/).

Two independent (replicate) experiments were performed using 22 multiparous Topigs-20 sows housed and inseminated at research facility Carus (Wageningen University & Research, The Netherlands). The new-born piglets inhabited with the sow and littermates till weaning (4 weeks of age), and received ear tags for individual identification and an iron injection, standard to pig husbandry practice. Two days after birth, twelve litters were provided with fibrous diet (early fed group or EF) in addition to sow’s milk and the remaining ten litters suckled mother’s milk only. Additional details about the treatment, housing and management have been described previously^16,17^.

### Personality test or backtest

At approximately 2 weeks of age, the piglets were subjected to a backtest as described previously^20^. Their response in this test, which is heritable^36–38^, reflects their preferred coping strategy, or coping style, which is considered a personality trait. Several studies, often studying the extreme pigs at either end of the population, have revealed links between the backtest response of piglets with neuroendocrine features, gene expression patterns and behavioural characteristics in later life, including behavioural flexibility^39–41^. Briefly, in the backtest, piglets are manually restrained by putting them on their back (supine position) for 60 s. The recorded parameters during the test were: (1) latency until the first struggling attempt (latency_resist); (2) the total number of struggling attempts (frequency_resist); (3) latency until the first vocalisation (latency_vocalise); (4) the total number of vocalisations (frequency_vocalise).

### Combined open field and novel object test (or novel environment test)

At 3.5 weeks of age, a subset of piglets (n = 47) was subjected to a 10 min combined open field test (OFT) and novel object test (NOT) in both the experiments (experiment 1, n = 19; experiment 2, n = 28 piglets). The selected piglets were balanced for pen, gender as well as average body weight of the litter at 21 days of age. Testing was carried out on two consecutive days and the order of piglets tested was balanced for gender and treatment. Fear-related behaviour and exploration were assessed using multiple behavioural observation scores obtained during the combined OFT and NOT, which have previously been described ^42^. The unfamiliar (or novel) environment, which was an arena of 3 × 3 m, with walls of 1.2 m and a concrete floor, was located in a test room at the end of the hallway, away from the home pen (visually and auditorily). The individual piglets were transported to the arena using a transport cart. Each piglet was placed in one corner of the test arena, in the same start position next to the wall. The pigs were given a 5-min period to explore the novel environment (OFT). After 5 min, a novel object (metal bucket) was slowly lowered from the ceiling into the centre of the arena until it touched the floor which, consequently, resulted in a noise. The piglets were given another 5 min to interact with the novel object (NOT). Behaviour and vocalisations (Supplementary Table 1) were continuously scored live using Psion hand-held computers with the Pocket Observer 3.1 software package (Noldus Information Technology, Wageningen, The Netherlands). Two mutually exclusive behavioural classes were observed simultaneously, one recording the posture or locomotion of the piglet as states, and the other recording the (other) behavioural states displayed by the piglet whilst in a particular posture or locomoting.

### Sampling, DNA extraction and 16S rRNA gene based amplicon sequencing

For microbiota analysis, rectal swab samples were collected at 2 and 4 weeks of age, by inserting a sterile cotton swab (Puritan Medical, Guilford, ME USA; Cat Number-25-3306-U) 20–30 mm into the rectum and rotating the swab against the bowel wall for a minute before placing it into a 5 ml Eppendorf tube. The samples were kept on ice during transport to the laboratory and stored at − 20 °C until further processing. All pigs (except one) that participated in the behavioural tests were also sampled for microbiota. At 2 weeks of age, the back test was performed, followed by microbiota sampling the next day. At 3.5 weeks of age, the OFT/NOT was done in 2 consecutive days, which was followed by microbiota sampling at 4 weeks of age.

As previously described in detail^16^, DNA extraction was performed by the repeated bead beating method^43^ using QIAamp DNA Stool Mini Kit (Qiagen, Hilden, Germany). Briefly, the DNA template was used to amplify the V3-V4 region of the bacterial 16S rRNA gene, purified and subsequently sequenced using (paired-end) Illumina MiSeq system at BaseClear BV (Leiden, The Netherlands). After quality filtering, the Illumina reads were imported into the CLC Genomics Workbench version 11.01, processed using the CLC Microbial Genomics Module version 2.5.1 (CLC bio, Arhus, Denmark) and the high quality sequences were finally clustered into operational taxonomic unit (OTUs) at 97% identity threshold using SILVA database v132^44^.

### Correlation analysis

While combining the datasets, we had to omit one pig as it was not selected in the behavioural tests. The backtest (at 2 weeks of age), reflecting a pig’s coping style or personality, was analysed with microbiota information obtained from rectal swabs taken at the same timepoint (2 weeks of age), whereas the novelty test observations (at 3.5 weeks of age; related to anxiety, fear and exploration) were assessed with microbiota data collected at 4 weeks of age (Fig. 1). To evaluate associations between microbiota composition (genus level) and behavioural variables, multivariate redundancy analysis was performed in CANOCO 5 software (Microcomputer Power, Ithaca, NY, USA)^45^. Redundancy analysis (RDA) is a canonical version of principal component analysis where the principal components are constrained to be linear combinations of the explanatory variables. RDA is a type of constrained ordination that assesses how much of the variation in response variables (here, microbial taxa) can be explained by the variation in explanatory variables (here, behavioural observations), in addition to providing related microbial taxa with the explanatory variable. Partial redundancy analysis (pRDA) was employed in this study to analyse the associations between microbiota and behavioural variables, after experiment and treatment were removed (partialled out) from the ordination. Statistical significance was assessed by the Monte Carlo permutation procedure (MCPP) with 499 random permutations. The behavioural observations (Supplementary Table 1) were categorised per test, i.e. backtest, OFT, NOT, and analysed separately. Some of the behavioural observations were summed to obtain additional parameters, for example, frequency of squeals, grunt squeals and screams were summed as ‘high pitched vocalisations’. Similarly, ‘exploring behaviour’ was obtained by summing nosing and rooting behaviour on the walls and floor of the test arena. Lying did not occur and sitting was very rare and displayed by only a number of piglets, and were not included in the analyses. Frequency of excretions was not included in the analyses, as it was found to be distant from the mean of coefficient of variation (CV%) (Supplementary Fig. 1A). We employed relatively permissive cut-off values in the RDA analyses to identify potential microbiota associations, where behavioural parameters having *P* value < 0.1 were considered significant and the associated microbial groups (genus level), having (1) a minimum response scores ≥ 0.35 and (2) 0.1% relative abundance in at least 10% of samples, were considered relevant. The associations between individual behavioural scores and microbial groups (identified in pRDA) were subsequently tested by non-parametric Spearman correlation analysis (*P* < 0.05 considered significant) in GraphPad Software 8.1.1 (California, USA, www.graphpad.com).

In addition, Multivariate Analysis by Linear Models (MaAsLin)^46^ was employed in the galaxy platform (https://huttenhower.sph.harvard.edu/galaxy/) to further test the significant behavioural observations and their associated microbial taxa. MaAsLin is a multivariate statistical linear regression analysis that identifies associations between metadata and microbial community abundance. MaAsLin allows detection of the effect of one variable without the influence of other metadata in the study. Initially, the default MaAsLin parameters were applied (minimum 0.01% relative abundance present in 1% of samples, *P* < 0.05, FDR < 0.05), but to more accurately compare MaAsLin results with redundancy analyses, subsequent MaAsLin analyses employed taxa representing ≥ 0.1% of the total microbial composition prevalent in > 10% of all samples.

## Supplementary Information

Supplementary Information 1.

Supplementary Information 2.

## Data Availability

Raw sequences can be found on SRA-NCBI (Sequence Read Archive-National Center for Biotechnology Information) database under the SRA accession number PRJNA684557 (experiment 1; day 15 and day 28), PRJNA811176 (experiment 2; day 15) and PRJNA775018 (experiment 2; day 28) (more in Supplementary_data_information).
