# Supplementary material for: Exploring the association between microbiota and behaviour in suckling piglets
Source: Sci Rep. 2022 Jul 19;12:12322. doi: 10.1038/s41598-022-16259-3 (PMC9296644; doi:10.1038/s41598-022-16259-3)
Supplement: Supplementary file 1 — Supplementary Information 1. [file 41598_2022_16259_MOESM1_ESM.pdf]

Supplemental information belonging with:

Exploring the association between microbiota and behaviour in suckling piglets

R. Choudhury, A. Middelkoop, J.E. Bolhuis, M. Kleerebezem

Supplementary table 1: Ethogram of behavioural states and vocalisations scored in the combined open field and novel object test. Behavioural states were scored in two mutually exclusive classes: "Locomotion and postures" and "Behaviour". The behaviours mentioned under "novel object test only" were added to the "Behaviour" class for that phase of the test.

**Behaviour**	**Definition**
Open field test/ Novel object test	
**Locomotion and postures**	
Walking	All four legs move or the piglet turns around at the same spot without moving all four legs
Standing	Standing with four paws on the floor without moving them, excluding standing alert
Standing alert	Standing motionless with ears upright and head fixed (up or down)
Sitting or lying	Sitting or lying on the floor without performing any other described behaviour
**Behaviour**	
**Exploring floor**	
Nosing floor	Sniffing, touching (with snout) or licking the floor. Rooting disc is either in contact or very close to the floor
Rooting floor	Rooting the floor with the rooting disc that exerts some force
**Exploring walls**	
Nosing walls	Sniffing, touching (with snout) or licking the walls of arena. Rooting disc is either in contact or very close to the surface
Rooting walls	Rooting the walls of arena with the rooting disc that exerts some force
**Low-pitched vocalisations (events)**	
Short grunt	A low tone of less than half a second (one note)
Long grunt	A low tone of more than half a second (one note)
**High-pitched vocalisations (events)**	
Squeal	A high tone (different notes)
Grunt-squeal	A low tone that transforms into a high tone
Scream	A high, long and loud tone, often as long as an expiration
Novel object test only	
Draw back	Drawing back from the novel object (NO; bucket) by walking away from the object or turning around at the same spot. At first the head is directed to the object, during the draw back the head can be either directed to the object or turned away from it
**Approaching novel object**	
Slow approach novel object	Approaching the novel object slowly (step by step) within 1m distance
Fast approach novel object	Approaching the novel object quickly (easy walking or running) within 1m distance
**Exploring novel object**	
Touch novel object	Sniffing, touching (with snout), rooting or licking the novel object. Rooting disc is in contact with the object
Explore novel object	Sniffing the novel object without touching it. Rooting disc is not in contact with the object
Chew novel object	Touching novel object with open mouth and making biting movements
Latency to explore novel object	Latency until the time the piglet either touched or explored (sniffing without touching) the novel object

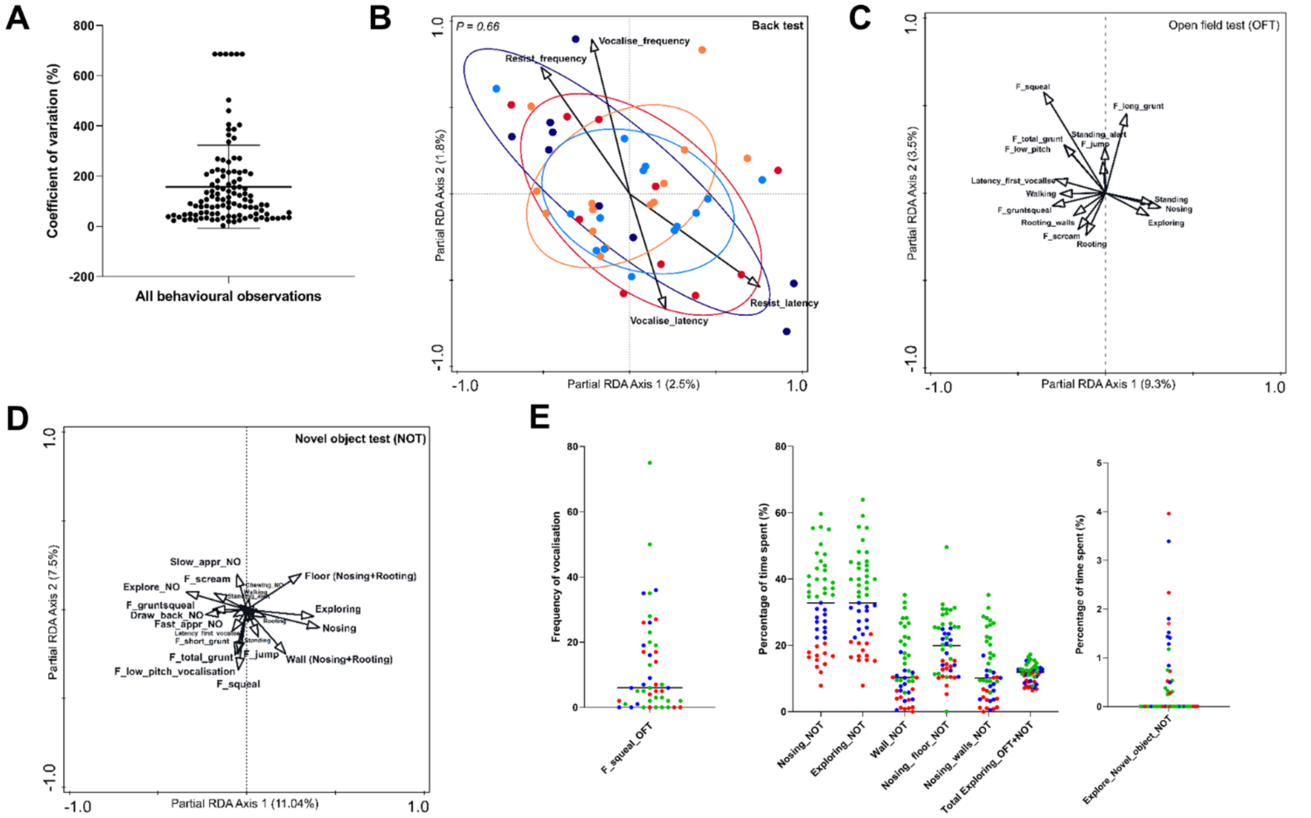

Supplementary figure 1: (**A**) Coefficient of variation (CV%) of all the behavioural variables observed in the backtest, combined open field test (OFT) and novel object test (NOT). (**B**) Partial redundancy analysis (pRDA) of the personality or backtest showing individual piglets belonging to exp1_EF (red), exp1_CON (dark blue), exp2_EF (orange) and exp2_CON (light blue) groups. (**C**) pRDA of all the variables in OFT. (**D**) pRDA of all the variables in NOT. The arrow length represents the strength of the correlation between the environmental variables and the microbes. The longer the arrow length, the stronger the correlation. (**E**) Scatter plot of the individual behavioural variables showing the spread of the data. Based on the 'nosing behaviour' quartiles, the individual piglets were divided into three groups: High (green; maximum to median), moderate (blue; median to 25th percentile) and low (red; 25% percentile- minimum) nosing behaviour. The other behavioural parameters were visualised using this classification.

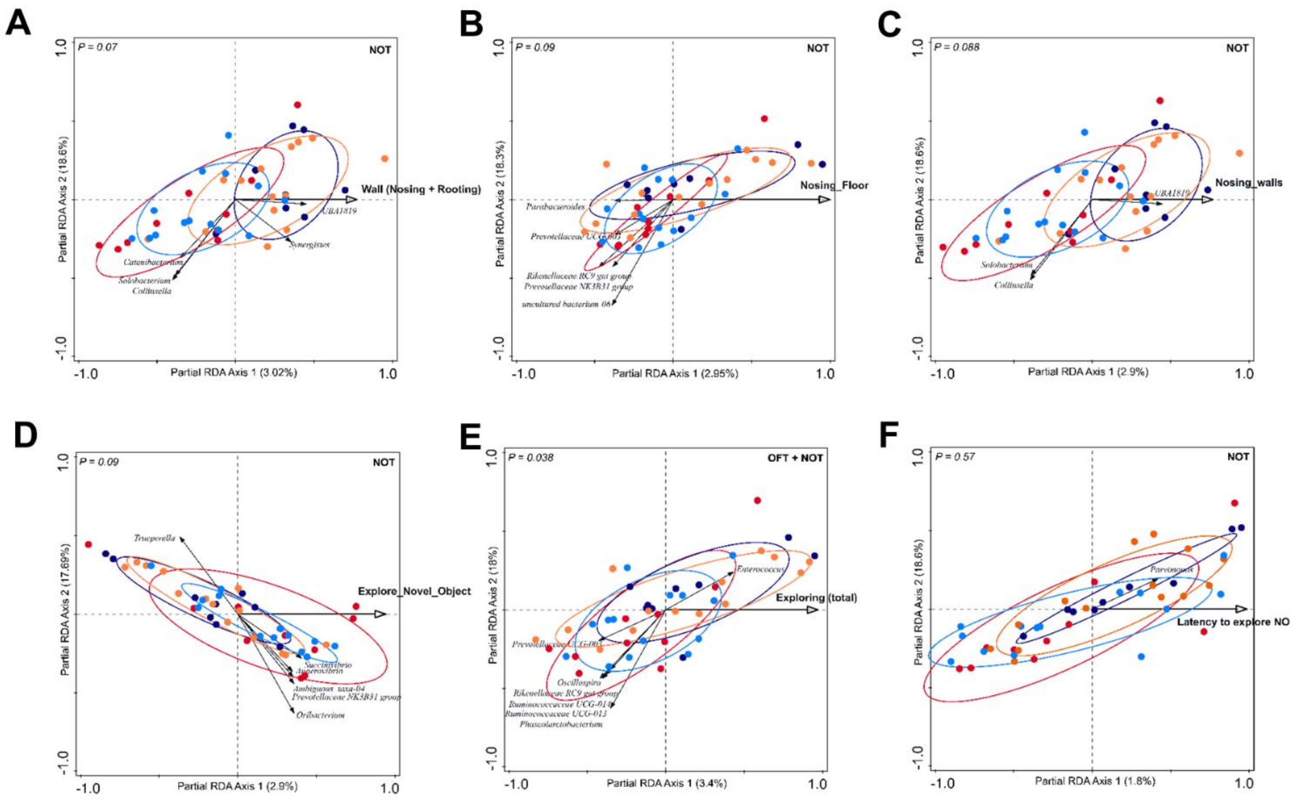

Supplementary figure 2: Partial redundancy analysis of the behavioural variables (corrected for experiment and treatment), with a P < 0.1. (**A**) Wall (nosing + rooting behaviour on the walls) in novel object test (NOT). (**B**) Nosing_floor (nosing behaviour in the floor only) in NOT. (**C**) Nosing_walls (nosing_behaviour on the walls only) in NOT. (**D**) Exploring_novel_object (exploring the bucket) in NOT. (**E**) Exploring_total (total exploring behaviour: nosing + rooting in both floor and walls) in combined OFT and NOT. (**F**) Latency to explore NO (novel object) in NOT.

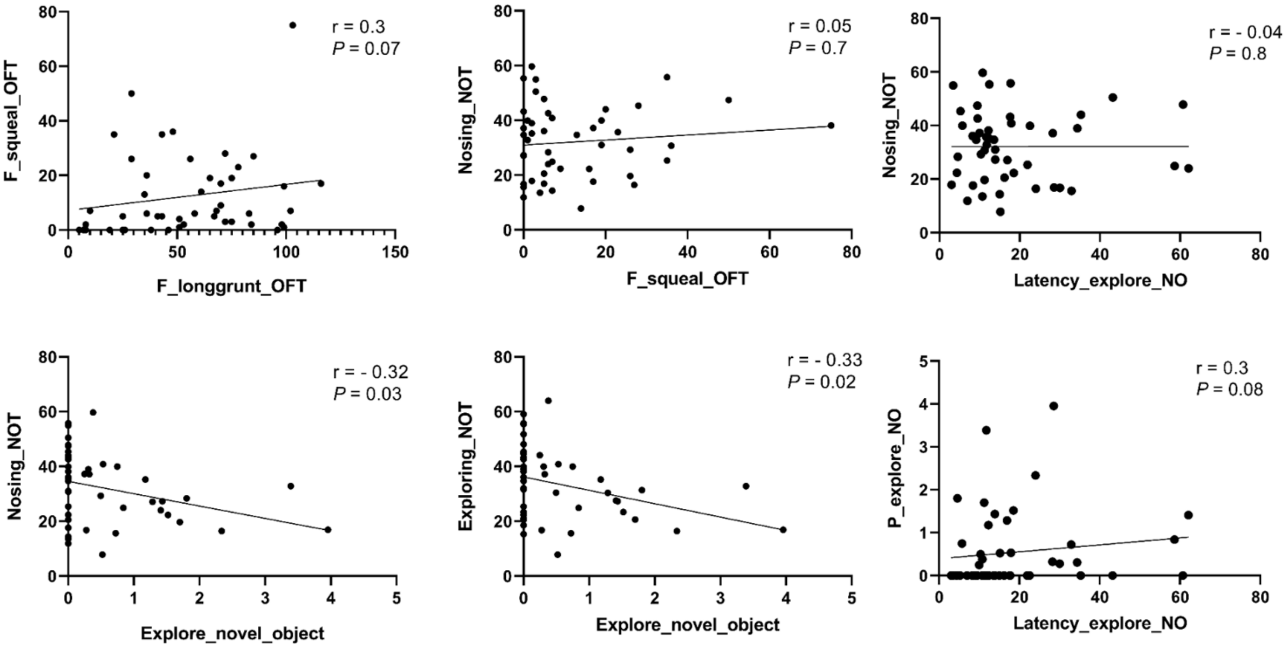

Supplementary figure 3: Spearman correlation analysis between different behavioural parameters: vocalisations ("squeal" and "long grunt" in OFT); vocalisation and exploring ("squeal" in OFT and "nosing" in NOT); exploration directed towards novel object ("explore NO") and general exploration ("nosing" and "exploring" during NOT); latency to explore NO and general exploration ("nosing"); latency to explore NO and explore NO.

Supplementary table 2: Behavioural responses observed in the combined open field test (OFT) and novel object test (NOT) at 3.5 weeks of age in suckling piglets (n = 47), expressed as Mean ± SEM. The variables with a prefix "F" denotes frequency of that event and "P" denotes the percentage of time (%) spent on that behavioural state.

**Open field test (OFT)**			**Novel Object test (NOT)**	
**Variable**	**Mean±SEM**		**Variable**	**Mean±SEM**
F_shortgrunt	113 ± 9.2		F_shortgrunt	121 ± 8.8
F_longgrunt	55 ± 4.3		F_longgrunt	48 ± 3.9
F_bark	0.1 ± 0.1		F_bark	0.7 ± 0.4
F_lowpitch	168 ± 8.7		F_lowpitch	170 ± 8.3
F_squeal	12 ± 2.2		F_squeal	20 ± 3.1
F_gruntsqueal	31 ± 6.0		F_grunt_squeal	22 ± 4.7
F_scream	2.3 ± 1.3		F_scream	2 ± 0.7
F_highpitch	45 ± 7		F_highpitch	44 ± 5.7
F_jump	2.2 ± 0.45		F_jump	3 ± 0.6
P_walking	48 ± 1.9		P_walking	35 ± 1.8
P_standing	50 ± 1.7		P_standing	61 ± 1.8
P_standing_alert	1.9 ± 0.4		P_standing_alert	3 ± 0.5
P_nosing_floor	42 ± 2.2		P_nosing_ floor	19.5 ± 1.3
P_rooting_floor	1.7 ± 0.5		P_rooting_floor	1.2 ± 0.3
P_nosing_walls	14 ± 0.9		P_nosing_walls	13 ± 1.4
P_rooting_walls	0.39 ± 0.1		P_rooting_walls	0.2 ± 0.1
Nosing	56 ± 2.2		Nosing	32 ± 1.9
Rooting	2.1 ± 0.6		Rooting	1.4 ± 0.4
Floor	44 ± 2.4		Floor	21 ± 1.4
Wall	14 ± 0.9		Wall	13 ± 1.4
Exploring	58 ± 2.4		Exploring	34 ± 1.9
			P_draw_back_NO	0.3 ± 0.1
			P_slow_approach_NO	0.6 ± 0.23
			P_fast_approach_NO	1.0 ± 0.1
			P_touch_NO	13 ± 1.5
			P_explore_NO	0.5 ± 0.13
			P_chewing_NO	1.2 ± 0.39
			Latency_explore_NO	18 ± 2.1

Supplementary table 3: Correlation of behavioural variables with microbial taxa having 0.1% relative abundance in at least 10% of samples, using MaAsLin Analysis in Galaxy (Huttenhower lab). The variables listed here have been identified in both MaAsLin ($P < 0.05$) and redundancy (RDA) analysis ($P < 0.1$), along with the corresponding regression coefficient, number of observations, number of non-zero observations, P-value, and Q-value (FDR-adjusted P-value using Benjamini–Hochberg) determined by MaAsLin analysis. The microbial taxa marked in deep green (response score ≥ 0.35), light green (response score ≥ 0.1) represent taxa that are identified in both the analysis. The light red coloured taxa indicate associations identified in RDA in opposite direction.

Variable	Microbial genera	Coefficient	N	N.not.0	P-value	Q-value
F_squeal_OFT	Bacteria|Firmicutes|Clostridia|Clostridiales|Ruminococcaceae|Anaerotruncus	0.0003	47	9	0.012	1.00
F_squeal_OFT	Bacteria|Firmicutes|Clostridia|Clostridiales|Lachnospiraceae|Oribacterium	0.0017	47	41	0.013	1.00
F_squeal_OFT	Bacteria|Bacteroidetes|Bacteroidia|Bacteroidales|Rikenellaceae|Alistipes	0.0009	47	32	0.020	1.00
F_squeal_OFT	Bacteria|Bacteroidetes|Bacteroidia|Bacteroidales|Muribaculaceae|CAG-873	0.0041	47	41	0.020	1.00
F_squeal_OFT	Bacteria|Proteobacteria|Gammaproteobacteria|Pasteurellales|Pasteurellaceae|Actinobacillus	0.0010	47	35	0.024	1.00
F_squeal_OFT	Bacteria|Bacteroidetes|Bacteroidia|Bacteroidales|uncultured|uncultured bacterium	0.0008	47	31	0.028	1.00
F_squeal_OFT	Bacteria|Synergistetes|Synergistia|Synergistales|Synergistaceae|Pyramidobacter	0.0009	47	28	0.040	1.00
F_squeal_OFT	Bacteria|Cyanobacteria|Melainabacteria|Gastranaerophilales|Clostridium sp. K4410.MGS-306|Clostridium sp. K4410.MGS-306	0.0004	47	11	0.043	1.00
Nosing_NOT	Bacteria|Proteobacteria|Deltaproteobacteria|Desulfovibrionales|Desulfovibrionaceae|uncultured	-0.0015	47	20	0.003	1.00
Nosing_NOT	Bacteria|Firmicutes|Clostridia|Clostridiales|Family XI|W5053	0.0019	47	27	0.005	1.00
Nosing_NOT	Bacteria|Firmicutes|Clostridia|Clostridiales|Lachnospiraceae|Syntrophococcus	-0.0064	47	7	0.007	1.00
Nosing_NOT	Bacteria|Firmicutes|Clostridia|Clostridiales|Lachnospiraceae|[Eubacterium] hallii group	-0.0141	47	43	0.024	1.00
Nosing_NOT	Bacteria|Firmicutes|Clostridia|Clostridiales|Family XIII|Mogibacterium	-0.0081	47	41	0.032	1.00
Nosing_NOT	Bacteria|Firmicutes|Clostridia|Clostridiales|Lachnospiraceae|[Eubacterium] eligens group	-0.0015	47	10	0.032	1.00
Nosing_NOT	Bacteria|Firmicutes|Clostridia|Clostridiales|Family XIII|Family XIII UCG-001	-0.0006	47	39	0.039	1.00
P_explore_bucket_NOT	Bacteria|Bacteroidetes|Bacteroidia|Bacteroidales|Prevotellaceae|Prevotellaceae UCG-001	0.0172	47	27	0.000	0.87
P_explore_bucket_NOT	Bacteria|Firmicutes|Clostridia|Clostridiales|Lachnospiraceae|[Eubacterium] eligens group	0.0079	47	10	0.002	1.00
P_explore_bucket_NOT	Bacteria|Bacteroidetes|Bacteroidia|Bacteroidales|Prevotellaceae|Prevotella 9	0.0526	47	45	0.003	1.00
P_explore_bucket_NOT	Bacteria|Bacteroidetes|Bacteroidia|Bacteroidales|Prevotellaceae|Prevotellaceae NK3B31 group	0.0269	47	45	0.014	1.00
P_explore_bucket_NOT	Bacteria|Cyanobacteria|Melainabacteria|Gastranaerophilales| Ambiguous_taxa| Ambiguous_taxa	0.0051	47	23	0.015	1.00

P_explore_bucket_NOT	Bacteria|Firmicutes|Clostridia|Clostridiales|Lachnospiraceae|Coprococcus 2	0.0096	47	11	0.042	1.00
P_explore_bucket_NOT	Bacteria|Firmicutes|Clostridia|Clostridiales|Lachnospiraceae|Coprococcus 1	0.0178	47	39	0.046	1.00
P_explore_bucket_NOT	Bacteria|Firmicutes|Clostridia|Clostridiales|Ruminococcaceae|CAG-352	0.0060	47	10	0.047	1.00
P_explore_bucket_NOT	Bacteria|Bacteroidetes|Bacteroidia|Bacteroidales|Prevotellaceae|Alloprevotella	0.0171	47	47	0.048	1.00
P_nosing_ floor_NOT	Bacteria|Firmicutes|Clostridia|Clostridiales|Family XIII|Mogibacterium	0.0092	47	41	0.023	1.00
P_nosing_ floor_NOT	Bacteria|Bacteroidetes|Bacteroidia|Bacteroidales|uncultured bacterium|uncultured bacterium	0.0019	47	7	0.036	1.00
P_nosing_ floor_NOT	Bacteria|Firmicutes|Clostridia|Clostridiales|Lachnospiraceae|[Eubacterium] hallii group	0.0144	47	43	0.036	1.00
P_nosing_walls_NOT	Bacteria|Firmicutes|Erysipelotrichia|Erysipelotrichales|Erysipelotrichaceae|Erysipelotrichaceae UCG-007	-0.0045	47	27	0.030	1.00
P_nosing_walls_NOT	Bacteria|Elusimicrobia|Elusimicrobia|Elusimicrobiales|Elusimicrobiaceae|Elusimicrobium	0.0010	47	27	0.033	1.00
P_nosing_walls_NOT	Bacteria|Proteobacteria|Deltaproteobacteria|Desulfovibrionales|Desulfovibrionaceae|uncultured	0.0007	47	20	0.035	1.00
P_nosing_walls_NOT	Bacteria|Fusobacteria|Fusobacteriia|Fusobacteriales|Fusobacteriaceae|Fusobacterium	-0.0029	47	42	0.035	1.00
P_nosing_walls_NOT	Bacteria|Firmicutes|Clostridia|Clostridiales|Family XI|W5053	-0.0012	47	27	0.037	1.00
P_nosing_walls_NOT	Bacteria|Firmicutes|Clostridia|Clostridiales|Defluviitaleaceae|Defluviitaleaceae UCG-011	-0.0006	47	39	0.037	1.00
P_nosing_walls_NOT	Bacteria|Actinobacteria|Actinobacteria|Corynebacteriales|Corynebacteriaceae|Corynebacterium 1	0.0025	47	44	0.039	1.00
P_nosing_walls_NOT	Bacteria|Synergistetes|Synergistia|Synergistales|Synergistaceae|Cloacibacillus	0.0005	47	30	0.039	1.00
P_nosing_walls_NOT	Bacteria|Firmicutes|Clostridia|Clostridiales|Ruminococcaceae|CAG-352	0.0004	47	10	0.040	1.00
P_nosing_walls_NOT	Bacteria|Bacteroidetes|Bacteroidia|Bacteroidales|Muribaculaceae|Ambiguous_taxa	0.0019	47	34	0.044	1.00
Wall_NOT	Bacteria|Firmicutes|Clostridia|Clostridiales|Lachnospiraceae|[Eubacterium] fissicatena group	0.0050	47	41	0.049	1.00
Exploring_NOT	Bacteria|Firmicutes|Clostridia|Clostridiales|Lachnospiraceae|[Eubacterium] eligens group	0.0016	47	10	0.019	1.00
Exploring_NOT	Bacteria|Firmicutes|Clostridia|Clostridiales|Lachnospiraceae|Syntrophococcus	0.0045	47	7	0.030	1.00
Exploring_NOT	Bacteria|Firmicutes|Clostridia|Clostridiales|Lachnospiraceae|Marvinbryantia	-0.0041	47	46	0.039	1.00
Exploring_NOT	Bacteria|Firmicutes|Clostridia|Clostridiales|Lachnospiraceae|[Eubacterium] hallii group	0.0133	47	43	0.046	1.00
